# Exploring the impact of legislative absences on the working environment of gig workers in South Africa

**DOI:** 10.3389/fsoc.2026.1824697

**Published:** 2026-10-12

**Authors:** Zini Gwele, Calvin Mabaso, Xavier Knight

**Affiliations:** Department of Industrial Psychology and People Management, College of Business and Economics, University of Johannesburg, Auckland Park, South Africa

**Keywords:** algorithmic management, gig economy, job insecurity, legislative exclusion, platform capitalism, precarity, South Africa

## Abstract

This study pursues two objectives. Empirically, it examines how legislative exclusion is produced in South Africa and how gig workers experience it in their everyday working lives. Theoretically, it develops an interpretive account of the relationship between political and economic processes that are ordinarily treated as separate domains, reading political and regulatory exclusion as constitutive of the forms of economic insecurity and precarity that participants describe rather than as their determinant. The study is framed by the concept of legislative exclusion, understood as the process through which state action constitutes a category of workers as falling outside the scope of labor law; precarity, platform capitalism and algorithmic management are used to elaborate the processes through which that exclusion operates and the conditions with which it is associated. A qualitative phenomenological design was adopted. Semi-structured interviews were conducted with 10 purposively sampled gig workers in Johannesburg working across e-hailing, delivery, freelancing and content creation, and the data were analyzed using reflexive thematic analysis. The sample spans two structurally distinct segments of platform labor, which are analyzed separately throughout: location-based work (e-hailing and delivery), which is geographically tethered, capital-intensive for the worker and in principle regulable by the national state; and remote online work (digital freelancing and social media content creation), which is contracted across borders and mediated by clients and monetization programs rather than by dispatch algorithms. The analysis reads exclusion as actively produced rather than merely absent: a statutory definition of the employee that platform work is arranged to fall outside, a jurisdictional architecture that renders the responsible corporate entity difficult to reach, and a recent transport regulation that recognizes e-hailing while leaving the employment relationship untouched. These instruments do not bear on the two segments equally: the transport regulation reaches location-based drivers alone, whereas remote online workers are addressed by no domestic instrument at all. Exclusion is in turn bound up with unbounded working hours, exclusion from employment benefits and the absence of any accessible dispute-resolution forum, though it is lived differently in each segment. Location-based participants described unreviewable deactivation and the transfer of vehicle, fuel and licensing costs; online participants described client non-payment, unenforceable informal agreements and dependence on opaque monetisation and visibility rules. The study contributes an interpretive account in which legislative exclusion is understood as a structuring condition through which risk is transferred to workers, platform and client authority is rendered unreviewable, and disadvantage is compounded over time.

## 1 Introduction and background

The gig economy, defined as a labor market characterized by short-term, flexible jobs mediated through digital platforms, has transformed employment relations in South Africa (Tan et al., 2021). This form of work has become increasingly prominent as individuals seek alternative sources of income amid persistent unemployment, economic inequality, and labor market restructuring. Gig workers, typically classified as independent contractors, engage in services such as e-hailing, food delivery, and digital freelancing. These are not variants of a single occupational form. Following the distinction now standard in the field, this study separates location-based platform work, which is performed in a particular place for customers who are physically present and is therefore visible to, and in principle regulable by, the national state, from remote online work, which is performed for clients and audiences who may be located anywhere and is transacted through marketplaces, direct agreements or monetization programs governed by foreign terms of service (Graham et al., 2017; Heeks, 2017; Woodcock and Graham, 2020; Anwar and Graham, 2021; Keller, 2023). Legislative exclusion is common to both, but it is arrived at by different routes and encountered in different ways, and the two are kept analytically distinct throughout what follows. However, this classification places gig workers outside the scope of statutory labor protections afforded to employees under South African labor law.

In South Africa, the Labor Relations Act 66 of 1995 (LRA) regulates employment relationships by distinguishing between employees and independent contractors. Section 213 of the LRA defines an employee as a person who works under the direction or supervision of another, whereas independent contractors are regarded as autonomous service providers (LRA, 1995; Niselow v Liberty Life Association of South Africa Ltd, 1998). As a result, gig workers are excluded from fundamental protections, such as unfair dismissal safeguards, collective bargaining rights, and access to statutory dispute-resolution mechanisms. This single definitional boundary does the decisive work in the analysis that follows, and it is therefore set out once here rather than restated at each subsequent stage of the argument.

The expansion of the gig economy has been accelerated by structural economic shifts and technological advancement, with digital platforms facilitating new forms of labor organization (Woodcock and Graham, 2020). While flexibility is often presented as a key advantage of gig work, platform-mediated labor frequently involves algorithmic control, income volatility, and limited worker autonomy. Platforms are able to exercise control over task allocation, pricing, and performance evaluation without assuming the responsibilities traditionally associated with employment relationships (Wood et al., 2019). This asymmetry raises critical concerns regarding fairness, power, and vulnerability within platform–worker relations.

The South African setting gives these concerns particular force, and the case was selected on three grounds. The first is empirical. Unemployment is severe and structural rather than cyclical: in the first quarter of 2026 the official rate stood at 32,7 per cent, rising to 43,7 per cent on the expanded definition, with 3,9 million discouraged work-seekers and youth unemployment at 45,8 per cent among those aged 15 to 34 (Statistics South Africa, 2026). More than three-quarters of the unemployed have been so for a year or longer, and the country's Gini coefficient of approximately 0,63 is the highest recorded internationally (World Bank, 2022). These figures matter less as a description of hardship than as an account of labor supply: they point to a large population for whom platform work is not an alternative to employment but a substitute for it, so that the conditions of compulsion described below are characteristic of the labor market from which platform workers are drawn rather than incidental to the sample.

The second ground is analytical, and it is the substantive reason for the selection. Research on platform labor in the global South has converged on an account of regulatory absence: where the state has not legislated for platform work, workers are classified as independent contractors and fall outside the protective scope of labor law (Woodcock and Graham, 2020; Schor, 2020; Adams-Prassl, 2018). Wulansari et al. (2024) trace this process in Indonesia, where the absence of a protective framework leaves asymmetric algorithmic control beyond regulatory reach. The policy implication is straightforward: the remedy for absence is presence. The South African case unsettles this implication, because South Africa is not a case of regulatory absence. Section 23 of the Constitution entrenches a right to fair labor practices; the LRA and the Basic Conditions of Employment Act 75 of 1997 (BCEA) establish an extensive protective regime; sections 200A and 83A respectively install a rebuttable presumption of employment; the Code of Good Practice: Who is an Employee (2006) supplies an interpretive apparatus; and the Commission for Conciliation, Mediation and Arbitration (CCMA) provides accessible, low-cost adjudication. Platform workers stand outside every one of these instruments. Exclusion here is not intelligible as the absence of a regulatory state. It becomes intelligible, rather, in relation to the way a well-developed regulatory state draws and maintains the boundary of the employment relationship.

The third ground is historical. The use of the statutory definition of “employee” as an instrument of exclusion is not novel in this jurisdiction. Under successive Industrial Conciliation Acts, Black workers were excluded from the definition of employee and thereby from the machinery of collective bargaining and statutory protection. Exclusion is a characteristic technique of South African labor governance with a long institutional history. This is advanced as an analytical resonance rather than a direct institutional inheritance: it indicates that the boundary of the employment relationship has repeatedly been a site of political contestation here, which makes South Africa an unusually productive setting in which to examine how that boundary is drawn today.

The conditions that make platform work attractive in South Africa are themselves produced in part by state policy. Successive administrations have pursued labor market flexibility as a condition of insertion into global production networks, sustaining low-cost labor through externalized and intermediated employment relations while retaining formal protective standards for those who remain inside the employment relationship. The result is a labor market in which a shrinking protected core coexists with an expanding periphery of informalised work (Suwandi, 2019). The state's role is therefore twofold and continuous: it helps produce the conditions of economic compulsion that supply platform labor, and it declines to extend to that labor the protections it maintains elsewhere.

Against this background, the study addresses two questions. Empirically: how is legislative exclusion produced in South Africa, and how do gig workers experience it in relation to working conditions, job security, wellbeing and dispute resolution? Theoretically: how may that political exclusion be understood as constitutive of the economic insecurity participants describe, and how does that relationship differ between location-based and remote online platform work? The argument advanced is that legislative exclusion is not a regulatory vacuum awaiting technical correction but an actively maintained configuration of state practice, and that this configuration is the condition under which platform control comes to be experienced as precarity. The argument is offered as an interpretation of how participants make sense of their working lives rather than as a demonstration of law-like regularity; its warrant is analytical coherence with the accounts gathered, not predictive force.

The study proceeds as follows. Section 2 develops legislative exclusion as the analytical lens and situates precarity, platform capitalism and algorithmic management as accounts of the processes through which it operates and the conditions with which it is associated, and specifies the distinction between location-based and remote online platform work that organizes the empirical sections. Section 3 sets out the method. Section 4 analyses legislative exclusion as state practice and the way participants encountered it, distinguishing the two segments at each step. Section 5 analyses job insecurity as the form of experience associated with it, again by segment. Section 6 considers limitations, and Section 7 concludes.

## 2 The gig economy and legislative exclusion

This section combines what would conventionally be presented as a separate literature review and theoretical framework. It is organized as a single analytical sequence: legislative exclusion as the central organizing concept, the processes through which that exclusion operates, and the forms of insecurity and precarity with which it is associated.

The gig economy is commonly understood as a system of short-term, task-based work mediated by digital platforms, in which individuals are typically classified as independent contractors rather than employees (Anwar and Graham, 2019; Watson et al., 2021). While this classification affords platforms operational flexibility, it simultaneously excludes gig workers from labor legislation designed to protect employees, including minimum working conditions, collective bargaining rights, and social security benefits (Mokofe, 2022). Scholars argue that this legal classification is bound up with a marked asymmetry of power, in which platforms exercise control without corresponding accountability (Woodcock and Graham, 2020). In the South African context, gig work functions less as a voluntary flexible arrangement and more as a necessity-driven survival strategy, intensifying workers' exposure to insecurity and exploitation (Tan et al., 2021; Blom et al., 2023). Research conducted in Sub-Saharan Africa further underscores that the absence of formal protection mechanisms leaves gig workers vulnerable to low and unstable incomes, irregular work assignments, and limited opportunities for upward mobility (Ayentimi et al., 2025). This literature is drawn predominantly from location-based work, and the qualification of scope set out below indicates where its vocabulary extends to remote online work and where it does not.

The analytical lens adopted here is legislative exclusion. We use the term to denote the process by which state action constitutes a category of workers as falling outside the scope of labor law, and thereby outside entitlement to social protection, socio-economic rights and the machinery of collective representation. The concept rests on three assumptions drawn from the sociology of social exclusion. First, following Silver (1994), exclusion is relational rather than residual: it is not the absence of inclusion but an actively maintained boundary, sustained through institutional closure, which confers status on those within it precisely by withholding it from those outside. Second, the boundary is legal-definitional. In labor law the operative status is the employment relationship, and exclusion therefore operates through the definition of the employee rather than through any explicit act of exclusion, a form that renders it politically inexpensive because no group needs to be excluded by name. Third, exclusion is sustained by accommodation rather than by neglect. Following Novianto (2025), the state's institutional and political accommodation of platform capital, characterizing platform governance as a commercial matter and regulating the service while declining to regulate the relationship, sustains a cheap labor regime without requiring any positive decision to deprive workers of protection. Exclusion so understood is an accomplishment of the state rather than an omission by it.

Positioning legislative exclusion first, rather than as a supplement to theories of platform control, is a deliberate analytical choice with consequences for where explanatory emphasis is placed. An account that locates the origin of precarity in algorithmic management gives interpretive priority to the platform; the concept of legislative exclusion gives it to the state. The claim is not that the state produces precarity in a determinate or law-like fashion, but that the legal boundary of the employment relationship is the condition under which platform and client practices become intelligible to workers as beyond challenge. The three literatures discussed below are therefore introduced not as parallel or competing frameworks but as accounts of how exclusion is enacted and how it comes to be experienced.

The first concerns platform capitalism and the supplies business model. Srnicek (2017) characterizes platforms as a distinctive corporate form that extracts value by positioning itself as an intermediary between groups of users, and identifies “lean platforms,” those owning minimal fixed capital and outsourcing assets, costs and risks to workers, as the model on which most gig work rests. Vallas and Schor (2020) describe the platform as a “permissive potentate”: an organizational form granting workers considerable formal discretion over when and whether to work while retaining decisive control over the terms, pricing and continuity of that work. Adams-Prassl (2018) argues that this arrangement rests on a legal maneuver, namely the classification of workers as independent contractors, which allows firms to exercise employer-like control while disclaiming employer obligations. Van Doorn (2017) adds that the low-paid service work organized through platforms is disproportionately performed by women, migrants and racialized workers, so that the risks externalized by platforms fall along pre-existing lines of social inequality. Legislative exclusion is therefore not incidental to the platform business model but one of its conditions of possibility.

The second concerns algorithmic management and supplies the technology of control: the delegation of managerial functions such as task allocation, pricing, evaluation and discipline to software systems (Lee et al., 2015; Duggan et al., 2020). Rosenblat and Stark (2016) document pronounced informational asymmetries in these systems, while Kellogg et al. (2020) systematize how algorithms direct, evaluate and discipline workers. Veen et al. (2020) and Wood et al. (2019) report that algorithmic control combines wide temporal autonomy with tight outcome control, which they read as a paradox in which workers experience both freedom and intense pressure. Of particular relevance to the present study, algorithmic discipline, most obviously deactivation, resembles dismissal without attracting the procedural protections that attach to dismissal in law (Gandini, 2019; Wood and Lehdonvirta, 2021). Algorithmic management is accordingly positioned here as a process through which exclusion is enacted rather than as its point of origin: it is efficacious because it is unreviewable, and it is unreviewable because the relationship it governs is not recognized in law.

The third concerns precarity and supplies the vocabulary in which participants' accounts are read here. Precarity describes work that is uncertain, unstable and insecure, in which workers bear risks historically borne by employers or the state (Kalleberg, 2009). Standing (2011) argues that precarious workers form a distinct social category characterized not only by unstable earnings but by the loss of occupational identity, of enterprise-based benefits, and of the rights attached to secure employment; in his terms such workers become “denizens” rather than full citizens of the labor market. Bourdieu (1998) treats precarity as a mode of domination: insecurity disciplines workers by making them anticipate loss, so that compliance is secured without direct coercion. Kalleberg (2018) argues that the extent to which precarious employment is lived as a precarious life depends on the surrounding institutional architecture, which suggests that similar platform practices may be experienced differently across regulatory settings. Job insecurity is therefore treated in this study in relation to legislative exclusion rather than as a separate theoretical component.

Southern scholarship sharpens this point. Scully (2016) and Barchiesi (2011) caution that theories of precarity developed in the global North commonly assume a prior Fordist norm of stable, protected wage employment against which precarity appears as a departure. In South Africa, secure employment was never the general condition of the labor force: the labor market was historically structured by racialized migrancy, casualisation, and mass exclusion from formal work. Gig work therefore represents less a novel fall from security than the digital extension of a long-standing arrangement in which large numbers of workers labor beyond the protective reach of the law (Webster, 2020). Alberti et al. (2018) similarly argue that precarity is best analyzed as a historically and institutionally situated process rather than as a universal condition.

Flexible working arrangements are frequently cited as a defining feature of gig work, offering workers autonomy over hours and task selection (Watson et al., 2021). Under conditions of legislative exclusion, however, flexibility may become coercive rather than empowering, as workers are compelled to work extended hours to achieve financial stability (Mas and Pallais, 2020; Wood et al., 2019). In response, gig workers often develop informal coping strategies, including reliance on peer networks, off-platform negotiations with clients, and participation in online communities (Burtch et al., 2016; Van Belle et al., 2023). These practices reflect worker agency but also highlight the absence of formal institutional support, and scholars caution that they cannot substitute for legislative reform (Johnston and Land-Kazlauskas, 2018). The absence of platform cooperatives in South Africa is analytically informative in the same direction: cooperative alternatives are the product of sustained collective mobilization (Scholz, 2016; Sandoval, 2019), and the diversified, discontinuous and individually contracted relations produced by exclusion foreclose the sustained interaction on which collective identity depends (Standing, 1997; Suwandi, 2019). Their absence is thus a further aspect of the configuration this study seeks to interpret rather than evidence of worker preference.

A qualification of scope is necessary before these literatures are brought together, because platform labor is not a single occupational category and the vocabulary set out above does not apply evenly across it. Location-based platform work e-hailing and delivery is geographically tethered (Keller, 2023): it is performed in a particular city, for customers who are physically co-present, using capital equipment that the worker supplies, fuels and insures. It is consequently visible to the national state and susceptible to national regulation, as Section 4.1 illustrates, and the control exercised over it takes the classic algorithmic-management form of dispatch, rating, acceptance-rate discipline and deactivation. Remote online work digital freelancing and social media content creation is not tethered in this way. It is transacted through global marketplaces, direct client agreements or platform monetisation programmes governed by foreign terms of service; its counterparties are frequently located outside the jurisdiction; and its characteristic insecurities are non-payment, unpaid speculative labor, fee and exchange-rate deductions, and dependence on ranking, reach and monetisation criteria that are neither published nor contestable (Graham et al., 2017; Heeks, 2017; Wood et al., 2019; Anwar and Graham, 2021). Social media content creation sits at the far end of this continuum, since the creator's income derives from advertisers, brand partners and platform monetization rather than from an intermediary that allocates tasks, so that even the party against whom a claim might be directed is indeterminate. The three literatures accordingly illuminate the two segments unevenly Srnicek's lean-platform model and Kellogg et al. (2020) account of algorithmic control speak most directly to location-based work, whereas Graham et al. and Heeks's analyses of cross-border online outsourcing speak to the other and the analysis below reports the segments separately wherever participants' accounts diverge, treating what they share as something to be shown rather than assumed.

These literatures converge on the analytic proposition that organizes the analysis below: legislative exclusion is the condition under which platform control comes to be experienced as precarity. The state maintains the boundary, platform capitalism supplies the business model, algorithmic management and client discretion supply the modes of control, and precarity is the experience participants report at the end of that sequence rather than its point of departure. The sequence is offered as an interpretive ordering of the accounts gathered rather than as a chain of determination, and it holds for both segments only in the general form just stated; its specific content differs between them. This ordering locates analytic responsibility with the state rather than with the technology and identifies the object of any remedy as the scope of the employment relationship rather than the conduct of algorithms. Table 1 sets out the resulting analytical architecture.

**Table 1:** The analytical architecture of legislative exclusion in South African platform work.

Tier and analytical lens	Component	Operation and evidence in this study
Tier 1 — Structuring condition (State) Lens: social exclusion and the politics of exclusion (Silver, 1994; Novianto, 2025)	Definitional exclusion	s 213 LRA; dominant impression test; earnings-threshold limits on the s 200A and s 83A presumptions; applies to both segments
	Jurisdictional and corporate exclusion	Uber SA v NUPSAW (2018) 39 ILJ 903 (LC): exclusion produced through corporate structuring and jurisdiction rather than on the merits; more acute still for online work, where the counterparty is frequently a foreign client rather than a platform
	Regulatory displacement	National Land Transport Amendment Act: e-hailing regulated as public transport; licensing, branding and safety obligations imposed on the individual operator; employment relationship unaddressed; location-based work only — no comparable instrument addresses remote online work
	Discursive framing	Platform work framed officially as entrepreneurship and job creation; regulatory restraint presented as the protection of opportunity; framing applies to both segments, but is most fully naturalized for online freelancers and content creators
Tier 2 — Enacting processes (Platform and client) Lens: platform capitalism (Srnicek, 2017; Adams-Prassl, 2018; Vallas and Schor, 2020) and algorithmic management (Rosenblat and Stark, 2016; Duggan et al., 2020; Kellogg et al., 2020); for online work, reputational ranking and monetisation governance rather than dispatch (Graham et al., 2017; Heeks, 2017)	Algorithmic management	Job allocation, ratings, acceptance-rate discipline, deactivation without process or review (location-based); ranking, reach, search visibility and monetisation criteria (online)
	Contractual classification	Terms of service constituting the worker as an independent contractor of a foreign entity; for online work, frequently no written contract at all
	Cost transfer	Vehicle, fuel, maintenance, data, insurance and now operating-license costs borne by the worker (location-based); equipment, data, software subscriptions, unpaid pitching and platform fees (online)
	Unilateral variation	Commission rates and fare structures altered without notice, consultation or recourse; efficacious because unreviewable, and unreviewable because Tier 1 places the relationship outside legal cognisance; for online work, unilateral variation of ranking, reach and monetisation rules
Tier 3 — Experienced conditions (Worker) Lens: precarity (Bourdieu, 1998; Kalleberg, 2009, 2018; Standing, 2011) and the Southern critique of precarity (Barchiesi, 2011; Scully, 2016; Webster, 2020)	Temporal precarity	Working days extended well beyond statutory limits; no regulated rest periods or leave (S5.1); continuous availability across client time zones (online)
	Income precarity	Earnings volatile and net of transferred costs; no minimum wage floor (S5.1, S5.2); client non-payment and demonetisation (online)
	Social-protection exclusion	No UIF, no COIDA cover, no employer contribution to retirement or medical provision (S5.2)
	Procedural precarity	Deactivation functions as dismissal without any right to be heard or to refer a dispute (S4.2, S5.2); for online work, contract termination and non-payment with no forum in any jurisdiction
	Representational precarity	No recognized bargaining unit; no organizational rights; cooperative alternatives structurally foreclosed (S4.2)
	Physical precarity	Exposure to violence, road risk and long-hour fatigue borne individually (S5.3) (location-based); screen-based and psychological strain (online)

Tier 2 and Tier 3 entries are marked by segment where the two diverge. “Location-based” denotes e-hailing and delivery; “online” denotes digital freelancing and social media content creation.

Authors' elaboration.

## 3 Methodology

This study adopted an interpretivist qualitative research design to examine how gig workers experience and make sense of legislative exclusion in South Africa. An interpretivist paradigm was appropriate because the study seeks to understand subjective meanings and lived realities rather than measure variables or test predetermined hypotheses (Babbie and Mouton, 2001). A phenomenological strategy was employed, drawing on an interpretative (hermeneutic) rather than a descriptive Husserlian phenomenology: the researchers' interpretative work is treated as a resource for analysis rather than as a bias to be eliminated. This position is consistent with the reflexive thematic analysis described below and explains why the analysis moves beyond the description of experience to an account of the structural conditions in relation to which participants make sense of it.

Ethical clearance was obtained prior to data collection. Recruitment was concentrated in Johannesburg, the country's largest metropolitan labor market and the site of the densest platform activity. Purposive sampling was used to select participants with direct, lived experience of gig work, supplemented by snowball sampling to broaden access across platform sectors. Ten gig workers participated (aged 19 to 41; three men and seven women), working in e-hailing, delivery, digital freelancing and social media content creation (Table 2). Sampling was structured to span the two segments identified in Section 2: four participants undertook location-based work (three e-hailing drivers and one delivery courier) and six undertook remote online work (one freelance graphic designer and five social media content creators). The imbalance is stated here because it conditions how the findings should be read. Claims about location-based work rest on four accounts and are correspondingly more tentative than those about online work, and the two groups are nowhere aggregated in the analysis. Absolute figures rather than percentages are reported throughout, since proportions calculated on a sample of 10 would convey a misleading impression of precision. Prospective participants received an information sheet explaining the purpose of the study, and written informed consent was obtained prior to each interview.

**Table 2:** Sample profile.

P	Age	Gender	Education	Gig work and segment
1	34	Male	Matric	Uber driver (location-based)
2	19	Female	1st year college	Social media influencer (remote online)
3	38	Female	Matric	Social media influencer (remote online)
4	21	Female	Matric	Social media influencer (remote online)
5	32	Female	Bachelor's degree	Social media influencer (remote online)
6	34	Female	NQF 8	Mr Delivery driver (location-based)
7	37	Male	Matric	Uber driver (location-based)
8	34	Female	Matric	Freelance graphic design (remote online)
9	41	Male	Diploma	Bolt driver (location-based)
10	23	Female	Bachelor's degree	Social media influencer (remote online)

Authors' fieldwork.

Because the adequacy of a sample of 10 is likely to be questioned, the logic underpinning it is stated explicitly. The study does not claim data saturation. Saturation was developed within grounded theory and presupposes that meaning resides in the data awaiting exhaustion, whereas reflexive thematic analysis treats themes as analytic outputs generated through interpretative engagement, so there is no determinate point at which meaning can be declared complete (Braun and Clarke, 2019, 2021). Sample adequacy was instead assessed using Malterud et al. (2016) concept of information power. Each of its five dimensions supported a comparatively small sample here: the aim was narrow, concerning how the absence of statutory protection is experienced rather than the gig economy in general; the sample was densely specific, since every participant was actively engaged in platform work and directly subject to the exclusion under investigation; the analysis was theoretically informed; interview quality was strong, producing detailed and concrete accounts; and the analytic strategy was cross-case rather than case-by-case. Interviewing continued until the research team judged that every candidate theme was underpinned by accounts from several participants working across more than one platform sector, rather than by a single striking narrative. Because the sample spans two segments, information power was assessed within each rather than across the sample as a whole: candidate themes were retained only where supported by accounts from more than one participant in the segment to which they were attributed, and themes supported in one segment alone are reported as such rather than generalized to platform work at large.

Data were collected using semi-structured interviews, each lasting approximately 45 to 60 min. An interview guide was developed to align directly with the research focus, with core questions exploring how the absence of legislative protection shapes working conditions, job security and wellbeing, and probing questions designed to elicit concrete examples. The same core questions on working time, security, wellbeing and recourse were put to all participants, while the probes were adapted to the participant's segment, since the concrete instances to be elicited differ: dispatch, ratings, fares and deactivation in location-based work, and client agreements, payment, revisions, ranking and monetization in remote online work.

Interview transcripts were analyzed using reflexive thematic analysis following Braun and Clarke (2021) six-phase approach. The analysis was inductive, in that codes and themes were generated from the data rather than applied from a pre-existing coding frame; predominantly latent in orientation, in that it sought the assumptions and conditions underlying participants' accounts rather than only their surface content; and experiential in emphasis while retaining a critical edge (Braun and Clarke, 2006, 2022). Recordings were transcribed verbatim and read repeatedly alongside the audio with analytic notes; codes were generated systematically across the full dataset, capturing both semantic content (for example, “working sixteen-hour days,” “account suspended without warning”) and latent meaning (for example, “flexibility experienced as compulsion,” “complaining is dangerous”); codes were clustered into candidate themes on the basis of a shared central organizing concept rather than shared topic, and topic-based groupings were discarded on that ground; candidate themes were checked against their coded extracts and against the full dataset and retained only where supported by accounts from several participants across multiple sectors; and extracts were selected to evidence analytic claims rather than to speak for themselves. Themes are accordingly interpretative constructions rather than categories found in the data.

Consistent with reflexive thematic analysis, the researchers' positioning is treated as integral to the analysis rather than as a contaminant of it. The research team is located in a South African university and approached the study with a labor-relations orientation that regards statutory protection as a legitimate expectation of work; this commitment made the analysis attentive to exclusion and correspondingly carried the risk of underplaying the value participants attached to autonomy and to the accessibility of platform earnings. A reflexive journal was maintained throughout, and coding was discussed within the team in the role of “critical friends,” to test the coherence and richness of interpretations rather than to secure agreement between coders; consensus coding and inter-rater reliability statistics were not used because, within this paradigm, coder agreement is not an indicator of analytic quality (Braun and Clarke, 2019). Quality was assessed against criteria appropriate to this approach: whether each theme was built around a clearly stated central organizing concept, whether analytic claims were evidenced consistently across the dataset, whether extracts were interpreted rather than merely displayed, and whether the theoretical, methodological and analytic components were coherent with one another (Braun and Clarke, 2021).

Participation was voluntary and based on informed consent. Confidentiality was maintained through participant codes (P1–P10) and removal of identifying details, and audio files and transcripts were stored securely. Given the potential risks associated with platform work, particular care was taken to avoid identifying platforms, routes, locations or other details that could link participants to specific accounts.

## 4 Legislative exclusion as state practice

This section and the next present empirical evidence and theoretical interpretation together rather than separating findings from discussion. This section establishes the first term of the argument: that exclusion is politically and institutionally produced by the state and is encountered by workers as the absence of standing rather than as a right denied. Section 5 establishes the second: the forms of insecurity with which this exclusion is associated. Throughout both sections the two segments are treated separately. Where a process or an experience is specific to location-based or to remote online work this is stated, and where the accounts converge the convergence is shown rather than presumed.

### 4.1 How the state produces exclusion

Four processes are identifiable in the South African case, and together they support reading exclusion as an accomplishment rather than an omission. The first is definitional exclusion. The statutory definition of the employee in section 213 of the LRA, applied through the dominant impression test endorsed by the Code of Good Practice: Who is an Employee, together with the earnings-threshold limitation on the presumptions in section 200A of the LRA and section 83A of the BCEA, places platform work outside the protective scope by operation of ordinary interpretation rather than by any dedicated legislative act. This first process bears on both segments, though not identically. For location-based workers the counterparty against whom the definition would be tested is a platform operating locally; for online freelancers and content creators it is frequently an individual client or a foreign advertiser, so that the definitional question does not arise in a form that any South African forum could entertain.

The second is jurisdictional and corporate exclusion. In Uber South Africa Technology Services (Pty) Ltd v NUPSAW & Others, (2018) 39 ILJ 903 (LC), the Labor Court set aside a CCMA ruling that drivers were employees of the local entity, holding that the local and Netherlands-based entities had been improperly conflated, and expressly declining to determine whether the drivers were employees of the foreign parent. Exclusion in that instance was produced procedurally, through the question of which entity could be cited as respondent, without any determination on the merits of the relationship. The upshot is that the protective architecture remains formally intact while being practically unreachable. For remote online workers the difficulty is more acute still. Where the counterparty is a client in another country, or a monetization program operated by a foreign platform, there is often no local entity to cite at all. The jurisdictional obstacle that Uber SA presented as a question of corporate structure appears in online work as the ordinary condition of the relationship rather than as a contested feature of it.

The third is regulatory displacement, and it is the clearest illustration that exclusion is actively produced. The National Land Transport Amendment Act, gazetted in June 2024 and brought into operation in September 2025, recognizes e-hailing as a distinct category of public transport, requires operating licenses issued by Provincial Regulatory Entities, and imposes vehicle branding and panic-button obligations. Compliance costs fell on the individual driver-operator, and the Department of Transport has stated that other operational costs fall outside its purview. The employment relationship is nowhere addressed. This is regulation without recognition: the state constitutes the driver as a licensed transport operator and, in the same instrument, confirms that the driver is not a worker. The state thereby displays both the capacity and the willingness to regulate platform work, while confirming the register in which it will not do so. This process is, however, segment-specific, and the contrast is instructive. E-hailing is reachable by the state precisely because it is performed on South African roads: the state regulated what was visible to it and left untouched what was not. Remote online work has attracted no equivalent instrument. The freelancers and content creators in this study were not regulated in the wrong register; they were not addressed at all, which is a further modality of exclusion rather than the absence of one.

The fourth is discursive framing. Platform work is officially characterized as entrepreneurship, self-employment and job creation, a framing that positions regulatory restraint as the protection of opportunity in a context of mass unemployment, and that renders the extension of employment status a threat to livelihoods rather than protection of them. This framing reaches both segments, but it is not received in the same way. The location-based participants encountered it as a description imposed upon them, since they are visibly directed by a platform that sets fares and allocates work; the freelancer and the content creators described themselves in its terms, as brands and small businesses building an audience. For the online segment the entrepreneurial framing is therefore not only official but partly self-adopted, which makes the absence of protection correspondingly harder to name as exclusion rather than as the ordinary risk of running one's own concern.

These processes do not themselves produce long hours or income insecurity. They sustain a jurisdictional space in which platform practices operate unconstrained, and it is those practices, namely algorithmic allocation and rating, unilateral variation of commission and fare structures, deactivation without process, and the transfer of vehicle, fuel, data and now licensing costs to the worker, that are associated with the conditions participants describe in Section 5. The corresponding practices in remote online work are different in kind: client-set terms accepted without negotiation, payment released at the client's discretion, unpaid revisions and speculative pitching, and reach and monetisation determined by criteria the worker cannot inspect. What the two segments share is not the practice but the absence of any forum in which it may be contested.

### 4.2 Encountering exclusion: the absence of a forum

Participants' accounts of dispute resolution are presented here, rather than among the consequences analyzed in Section 5, because they evidence the workings of exclusion itself. Participants did not describe a forum that had failed them; they described the absence of any forum to which they understood they had standing. Deactivation operated as dismissal without any of the procedural incidents that attach to dismissal in law, and participants attributed this not to platform conduct alone but to their position outside the category of employee. This is legislative exclusion as it is encountered in practice: not the experience of a right denied, but the experience of never having been a bearer of the right. The form this took differed by segment. Location-based participants described the platform as the entity that judged them and against which no appeal lay; online participants described a more diffuse condition in which the counterparty was an individual client, while the platform, where one existed, positioned itself as a marketplace bearing no responsibility for the outcome. In the first case there is an addressee but no forum; in the second there is frequently neither.

Participants consistently described a sense of powerlessness when faced with unfair treatment, emphasizing that their exclusion from statutory bodies such as the CCMA leaves them with little to no recourse when disputes arise over payment, client complaints or account suspensions. These are not interchangeable idioms. Account suspension belongs to the location-based accounts and non-payment by clients to the online ones, as the extracts below indicate, and the two describe different absences: a decision that cannot be appealed in the first case, and an obligation that cannot be enforced in the second. As one participant explained:

“*There's no recourse for that kind of unfair treatment.” (Participant 1, e-hailing, location-based)*

Another highlighted how this exclusion limits the ability to negotiate or assert basic work-related claims:

“*It makes it difficult to negotiate terms and access certain benefits as well.” (Participant 2, content creation, remote online)*

In the absence of formal channels, many gig workers attempt to resolve disputes through direct negotiation with clients or through platform messaging systems. These interactions were frequently described as unequal and emotionally taxing:

“*I have to talk to the clients directly. It is always a difficult conversation because they hold all the power… sometimes I just have to agree.” (Participant 3, content creation, remote online)*

“*I usually try to resolve disputes through the platform's messaging systems and by contacting their support team. However, it can be a lengthy process, and I'm not satisfied with the outcome.” (Participant 5, content creation, remote online)*

The significance of these accounts lies in how the absence of dispute resolution bears on the distribution of power, rather than in the inconvenience it entails. Statutory dispute-resolution machinery does more than settle individual grievances: the availability of the CCMA alters what an employer may do in the first place, because decisions are taken in the knowledge that they may later be reviewed. Where that possibility is removed, platform and client decisions become final by default, and the worker's remaining options are acceptance or exit. Grievances that would be procedural matters in a regulated workplace are thereby converted into private losses, and the aggregate of those losses remains invisible: because there is no forum in which they are recorded, no evidentiary record accumulates that might support regulatory attention or collective claims. Legislative exclusion, in this sense, is doubly effective: it removes the remedy and it conceals the harm. This is consistent with research showing that algorithmic systems intensify procedural unfairness by enabling unilateral decisions without transparency or due process (Duggan et al., 2020; Tassinari and Maccarrone, 2020), but it locates the source of that unfairness in the legal status of the relationship rather than in the technology through which it is administered. This holds for both segments, but by different routes. For location-based participants the decision that could not be reviewed was the platform's; for online participants it was the client's, and the platform's terms of service positioned it as a facilitator rather than a party. The consequence is finality by default in each case, but the remedy implied is not the same, since a presumption of employment directed at platforms would reach the first group while leaving the second largely where it stands.

### 4.3 Exclusion under revision

The configuration described above is presently contested. On 26 February 2026, the Minister of Employment and Labor published two draft instruments in Government Gazette No. 54220: the Labor Law Amendment Bill, 2025 and the Labor Relations Amendment Bill, 2025, both following negotiations at the National Economic Development and Labor Council conducted between April 2022 and October 2024. Among the proposals are expanded statutory definitions of employer and employee, a strengthened presumption of employment capable of reaching workers on digital labor platforms, and a new framework governing on-call, 0 h and if-and-when arrangements. Were these provisions enacted in their present form, minimum wages, working-time regulation, social security, occupational health and safety coverage, and collective bargaining rights would, in principle, come within the reach of platform workers. That reach would not, however, be uniform across the two segments. A strengthened presumption of employment operates by identifying an employer, and it is platforms directing location-based work within South Africa that it would most readily reach. For the freelancer and content creators in this sample, whose counterparties are individual clients, advertisers and monetization programs situated outside the jurisdiction, the same presumption has no obvious respondent; and the proposed framework for on-call, zero-hours and if-and-when arrangements presupposes an engaging party of a kind that much remote online work does not have. Reform pursued exclusively through the employee definition would therefore narrow the exclusion described here for one segment while leaving the other substantially where it stands.

Three qualifications are necessary. First, the Bills are not law: the comment period closed on 28 March 2026 and the package remains in the pre-parliamentary phase, and South African labor legislation has historically moved slowly. Second, the package is a negotiated settlement rather than a straightforward extension of protection, since alongside the proposed expansion of the employee definition it curtails unfair dismissal recourse during probation and caps compensation for higher earners; reform of this kind is better read as a renegotiation of the boundary than as its dissolution, which is consistent with the account of institutional accommodation set out in Section 2. Third, comparable regulatory activity is underway elsewhere on the continent, and the International Labor Organization is engaged in standard-setting on platform work.

Two observations follow for the argument advanced here. First, the fact that reform is being pursued through the definition of the employee confirms the analysis: the boundary of the employment relationship is the operative site of both exclusion and any prospective inclusion, which is precisely what an account organized around regulatory absence would not readily anticipate. Second, the data presented in this study document lived experience at the close of a long period of exclusion, and therefore constitute a baseline against which the implementation of any such reform may subsequently be evaluated.

## 5 Job insecurity under legislative exclusion

This section analyses the forms of insecurity associated with the processes described above, reporting the two segments separately wherever participants' accounts diverge. It is organized around four dimensions of insecurity identified in the data: unbounded working time and physical risk; deactivation, disposability and the absence of benefits; informality and cumulative disadvantage; and the account read in comparative perspective.

### 5.1 Unbounded working time and physical risk

Participants consistently described harsh and demanding working conditions, which they directly linked to the absence of formal labor regulation. The accounts of the working day quoted immediately below are those of the e-hailing and delivery participants; the online participants described a working day that was long in a different sense, and their accounts are taken up separately at the end of this subsection. The most prominent issue was the normalization of excessively long working hours, with many reporting working up to 16 h a day to earn what they considered a “decent” income:

“*We can work for 16 hours or more just to make a decent income. You are a one-man show, expected to be ready every day to make money.” (Participant 1, e-hailing, location-based)*

These long hours were not framed as a choice or as flexibility, but as a necessity. Participants spoke about pushing themselves to work even when unwell, knowing that taking time off would result in immediate income loss:

“*Even when I had a high fever recently, I pushed myself to work because missing a day means losing income permanently.” (Participant 6, delivery, location-based)*

Fatigue emerged as a serious safety concern, especially for those in e-hailing and delivery services:

“*Fatigue builds up and is why drivers are frequently involved in accidents.” (Participant 9, e-hailing, location-based)*

Analytically, this pattern amounts to more than an account of hard work. The limits the BCEA places on ordinary hours, its requirements for rest periods, and its provisions for paid sick leave do not merely protect individual employees; they establish a socially guaranteed boundary around the working day and place part of the cost of a worker's illness on the employer and the state. Where that boundary is absent, the limit on working time is, on these accounts, set by the worker's body and by the level of earnings required to survive. Long hours in this context are therefore not a preference expressed through flexibility but a rational response to an unregulated piece-rate structure, a dynamic consistent with evidence that flexibility becomes coercive where workers lack income protection (Mas and Pallais, 2020; Wood et al., 2019) and that economic pressure combined with unregulated rest is associated with heightened occupational risk (Meng et al., 2024). This is the first point at which legislative exclusion can be seen operating as a structuring condition rather than as a mere gap: income insecurity and exposure to physical risk are, in participants' accounts, inseparable, and the cost of maintaining and reproducing the workforce is described as falling on workers and their households rather than on platforms.

Unbounded working time was reported in both segments, but it was not the same phenomenon. For the e-hailing and delivery participants, time was unbounded in a physically hazardous way: the hours were spent driving, fatigue accumulated in a moving vehicle, and the risk fell on the body and on other road users, as Participant 9′s account of accidents makes plain. For the freelancers and content creators, the boundary problem was one of availability rather than exposure. Work was performed at home, but client communication, revision requests and the demand for a continuous posting schedule extended across evenings and weekends and, where clients were abroad, across time zones; and a considerable proportion of the labor pitching, negotiating, and producing content that is never commissioned or never monetised is performed speculatively and remunerated not at all. Participant 2, a content creator, described straining “physically and emotionally” with no provision made for it, and Participant 6, a delivery courier, described working through fever; the two accounts register the same absence of a socially guaranteed boundary around the working day from either side of the divide, but the statutory provisions whose absence is felt are not the same in each case. Ordinary hours limits, rest periods and paid sick leave speak to the first group; the second would be reached, if at all, only by rules governing minimum payment, the enforceability of client agreements and the timing of settlement. The absence of a working-time floor is thus common to both; what that absence permits is not.

### 5.2 Deactivation, disposability and the absence of benefits

The location-based participants overwhelmingly associated job insecurity with their classification as independent contractors, expressing a strong sense of disposability in relation to account deactivations that occur without warning or due process. Deactivation in this form belongs to that segment alone; its functional equivalent in remote online work is taken up below:

“*We drivers are disposable. A customer complains, Uber acts fast with no warning, no hearing. We have no protection.” (Participant 1, e-hailing, location-based)*

“*Your career is tied to your vehicle and the app; if your car breaks down or your account is suspended, your career is over until you fix it.” (Participant 1, e-hailing, location-based)*

Participants in both segments highlighted the lack of employment benefits, including medical aid, paid leave and retirement savings. Without employer contributions or statutory requirements, they were left to self-insure against illness, injury and old age:

“*If I need treatment for a chronic illness, I cannot afford it because I do not have medical aid.” (Participant 4, content creation, remote online)*

“*Benefits like medical aid and provident funds don't apply to us. You have to save for it separately, but most times there's not enough to save.” (Participant 9, e-hailing, location-based)*

What these accounts describe is a structural arrangement rather than a series of unfortunate events. Access to work is held at the discretion of an entity that is not, in law, an employer, and can be withdrawn without reasons, notice or hearing; the worker's dependence on the platform is therefore total, while the platform's obligation to the worker is minimal. Deactivation is, in effect, a dismissal; classified as the termination of a commercial arrangement, it attracts none of the protections that a dismissal would attract. Algorithmic management renders the arrangement operable at scale and at low cost (Rosenblat and Stark, 2016; Kellogg et al., 2020), but it is the legislative boundary established in Section 4.1 that leaves it unaccountable. This asymmetry is not static in participants' accounts: since deactivation is always possible and never reviewable, participants described modulating their conduct in anticipation of it, accepting unprofitable trips, declining to complain, and absorbing unfair ratings. Insecurity in this sense may be read as a mode of discipline (Bourdieu, 1998), and what it shapes therefore extends well beyond the cases in which a worker is actually deactivated. The absence of employment benefits compounds it by privatizing the costs of illness, injury and old age. Read against Standing's (2011) account of the precariat, participants occupy the position of denizens: economically active and indispensable to the operation of the platforms, yet holding a diminished set of rights in the labor market they sustain.

The functional equivalent of deactivation in remote online work is neither singular nor announced. None of the freelancers or content creators described being removed from a platform. What they described was the withdrawal of work and of payment by clients, as in Participant 4′s account of an agreement with no written contract that ended with no money, together with dependence on ranking, reach and monetization rules that determine whether work is offered or seen at all. Three features distinguish this position from the driver's. First, the loss is incremental rather than categorical: income declines without any decision having been taken that could be identified, still less contested. Second, the counterparty is dispersed across many clients, so that the dependence Participant 1 described as total is, for online workers, distributed which reduces exposure to any single relationship while removing even the possibility of addressing a complaint to a single party. Third, the recourse that a presumption of employment would supply is correspondingly harder to construct, since there is often no candidate employer within the jurisdiction. Both groups are subject to authority they cannot review, but the location-based worker is subject to a platform that behaves as an employer without being one, whereas the online worker is subject to a market that behaves as a labor market without any party in it acknowledging that role. The absence of benefits, by contrast, was reported in almost identical terms across the sample: neither segment is covered by the UIF or by COIDA, and neither receives an employer contribution of any kind.

### 5.3 Informality and cumulative disadvantage

Beyond economic insecurity, participants described broader social and psychological vulnerabilities. The absence of formal contracts and legal protection often pushed workers into informal arrangements. These took a different form in each segment: for the location-based participants informality was a deliberate move off-platform to recover margin from a counterparty they could identify, whereas for the online participants it was the ordinary condition of the engagement, work undertaken with no written agreement at all:

“*With some frequent customers, you build a relationship and then negotiate trips outside the app to avoid Uber's commission.” (Participant 1, e-hailing, location-based)*

“*There was no physical contract, and then it came back to the situation of me not getting my money.” (Participant 4, content creation, remote online)*

Participants in both segments repeatedly returned to concerns about wellbeing, particularly the lack of support when ill or unable to work:

“*It is straining physically and emotionally, but it is something I have to push through on my own. There is no medical or social provision made for this.” (Participant 2, content creation, remote online)*

“*Without paid sick leave, I work even when I'm sick. Without unemployment insurance, I have no cushion if clients stop giving me jobs.” (Participant 6, delivery, location-based)*

These accounts indicate how legislative exclusion is bound up with the reproduction of inequality over time rather than simply registering disadvantage at a single point. Without payslips, contracts or verifiable income, participants cannot satisfy the documentary requirements of credit, insurance, tenancy and other institutions that presuppose formal employment; exclusion from labor law thus becomes exclusion from financial and social citizenship (Casalhay et al., 2025). The informal workarounds participants described demonstrate agency, but they are self-limiting, since they operate outside any enforceable framework and shift additional risk onto the worker (Burtch et al., 2016; Johnston and Land-Kazlauskas, 2018). The pattern participants described is cumulative: insecure earnings leave little to accumulate, the absence of documentation closes the routes through which insecure earnings might be stabilized, and dependence on continued platform work deepens. In a labor market where unemployment exceeds 30 per cent and gig work serves as a primary rather than a supplementary source of income (Blom et al., 2023), the option of refusing these terms is largely notional; the absence of realistic alternatives is itself part of what holds the configuration in place. The documentary problem is nevertheless shaped by segment. The drivers' earnings pass through a platform that generates a statement of some kind, which is at least a partial record even where lenders and landlords decline to accept it; the freelancers and content creators described income arriving from multiple clients and advertisers under informal arrangements, so that even a partial record is often unavailable, and Participant 4′s experience shows how readily the absence of documentation becomes the absence of payment.

This configuration does not operate evenly across the workforce. Research on platform labor has consistently shown that the risks externalized by platforms fall disproportionately on workers already disadvantaged by gender, race, migration status and prior labor market position (Van Doorn, 2017; Hunt and Samman, 2019). In this sample, however, segment and gender are confounded and cannot be disentangled: all three men undertook location-based work, all six online participants were women, and the only woman in the location-based group was the delivery courier. It is therefore not possible to say whether the differences described above follow from the type of work or from the social position of those performing it. Read as a difference between segments rather than between genders, the accounts indicate that online participants worked under arrangements in which payment depended on informal client agreements and non-payment was a recurrent risk, while location-based participants worked under arrangements whose principal exposures were physical danger and the capital outlay required for a vehicle. Both are forms of unprotected work, but they distribute risk differently, and any gendered reading of that difference is advanced here as a question for larger and better-stratified samples rather than as a finding of this study. Read in this light, the South African case lends support to Southern critiques of precarity (Barchiesi, 2011; Scully, 2016; Webster, 2020): platform work here is not the erosion of a previously secure standard but the technological reorganization of an older arrangement in which substantial parts of the workforce labored beyond the protection of the law.

### 5.4 The account in comparative perspective

Although this study is grounded in a single national context, reading across contexts helps to distinguish what is common to platform work from what is specific to South Africa. The experiential grammar reported by participants recurs with striking consistency across jurisdictions: studies of remote platform workers in Sub-Saharan Africa and Asia report the same combination of temporal autonomy and intense pressure to remain continuously available (Wood et al., 2019); ethnographic accounts of food-delivery work in the United Kingdom (Cant, 2019) and Australia (Veen et al., 2020) describe the same experience of algorithmic direction without recourse; deactivation without stated reasons is documented among drivers in the United States (Rosenblat and Stark, 2016); and studies in Bengaluru report comparable patterns of long hours and income volatility (Surie and Koduganti, 2016). This convergence lends weight to reading the conditions described as bound up with a business model and its legal architecture rather than with local labor market conditions alone. The comparative literature is itself uneven in this respect. The ethnographies of food delivery and e-hailing cited above concern location-based work, whereas Wood et al. (2019) study of remote workers in Sub-Saharan Africa and South-East Asia and Graham et al. (2017) account of digital labor and development concern the online segment. Read separately, they suggest that the convergence noted here is stronger within segments than across them.

How that architecture is lived, however, differs substantially by context. Kalleberg (2018) argues that the significance of precarious employment is inseparable from the surrounding institutional environment. In much of Western Europe, platform workers excluded from employment status nevertheless retain access to universal healthcare and relatively substantial social assistance, so that exclusion from labor law does not entail exclusion from the systems on which subsistence and health depend. In South Africa, unemployment above 30 per cent removes the exit option that gives “flexibility” much of its meaning elsewhere, and exclusion from statutory protection coincides with limited access to private medical cover. Participants' accounts of working while ill and of having no protection against income interruption should be read against this background: what the South African case adds to the literature is the particular configuration of a strong, constitutionally grounded labor law regime from which a growing segment of the workforce is nevertheless formally excluded. For online workers the comparison is complicated further, since the relevant regulatory space is not only national. A South African freelancer contracting with a client in Europe or North America is governed in practice by whichever terms of service and private dispute procedures the marketplace imposes, and by no labor law at all; the institutional environment that Kalleberg treats as national is, for this segment, partly extraterritorial.

Regulatory developments elsewhere indicate the range of responses available. In Uber BV v Aslam (2021) UKSC 5 the United Kingdom Supreme Court held that drivers were “workers” entitled to the minimum wage and paid leave, reasoning that the terms of the written contract alone could not answer the statutory question. Spain's “Riders' Law” (Ley, 2021) introduced a presumption of employment for delivery workers, and Directive (EU) 2024/2831 (2024) combines a rebuttable presumption of employment with substantive obligations of transparency and human review in algorithmic management. Other jurisdictions have created intermediate categories or sector-specific welfare arrangements, such as California's Proposition 22 and India's Rajasthan Platform Based Gig Workers (Registration Welfare) Act (2023), though comparative scholarship cautions that intermediate statuses may entrench a lower tier of rights rather than close the protective gap (Cherry and Aloisi, 2017; Dubal, 2017).

For South Africa, the European approach is of particular interest because its two elements, a rebuttable presumption of employment and enforceable duties of algorithmic transparency, correspond directly to the two problems participants described: an unresolved classification question and decisions taken about them without stated reasons. It is also notable that South African law is not without instruments of this kind. Section 200A of the LRA and section 83A of the BCEA already establish a rebuttable presumption of employment for persons earning below the prescribed threshold, and the constitutional right to fair labor practices in section 23 is not, by its terms, confined to employees. The gap identified in this study is therefore as much a question of the interpretation and enforcement of existing instruments as of the absence of new ones. These comparisons are offered as exploratory rather than conclusive, since the studies referred to were conducted in different labor markets, with different platforms, samples and methods. It should be said plainly, however, that a presumption of employment addresses one segment far better than the other. Aslam, the Riders' Law and Directive (EU) 2024/2831 each presuppose an identifiable platform acting as a de facto employer within the jurisdiction, which describes e-hailing and delivery but does not describe freelancing or content creation, where the counterparty is a dispersed set of foreign clients. For that segment the more plausible instruments lie elsewhere: in payment-protection and escrow obligations imposed on marketplaces, in the extension of social security on a basis other than employment status, and in transnational standard-setting of the kind the International Labor Organization is pursuing. One implication of distinguishing the segments is therefore that they call for distinct regulatory responses rather than a single one.

## 6 Limitations

This study has several limitations. First, the qualitative, interpretivist design means the data reflect participants' subjective meanings and experiences, which may vary across settings and over time. To reduce the risk of researcher influence, reflexivity was practiced throughout and interpretative decisions were documented, so that the researchers' assumptions were made visible rather than assumed to have been set aside (Braun and Clarke, 2019).

Second, the sample was small (*n* = 10) and drawn using purposive and snowball sampling, which may introduce network bias and limits generalization to all gig workers in South Africa. The study accordingly makes no claim to statistical generalization; its claims are analytic, concerning the way in which legislative exclusion is implicated in the conditions participants described, and the reach of that account in other settings is a matter for further empirical examination. Third, the sample is internally heterogeneous and unevenly composed: four participants undertook location-based work and six remote online work, and five of the latter six were social media content creators. Claims about e-hailing and delivery therefore rest on a narrow base, and content creation is over-represented relative to its likely share of platform labor in Johannesburg. Because segment and gender are confounded in the sample, differences reported between the segments cannot be separated from differences of social position. Fourth, the study was conducted in Johannesburg and includes a specific mix of gig roles, so findings should be interpreted as context-specific rather than nationally representative. Fifth, it relied solely on self-reported accounts and did not include perspectives from platform companies, clients, unions, regulators or dispute bodies.

A sixth limitation is temporal: fieldwork was conducted before the commencement of the amended transport regulations and before publication of the proposed labor law amendments discussed in Section 4.3, so the accounts describe the regulatory configuration as it stood prior to those developments. Finally, the international parallels drawn in Section 5.4 and the observations offered about the gendered distribution of risk are advanced as exploratory interpretations rather than as demonstrated claims, since a sample of this size cannot support conclusions about the relative position of groups within it.

## 7 Conclusion and implications

This study set out to develop an interpretive account of the relationship between political exclusion and economic insecurity in South Africa's gig economy. Drawing on interviews with ten platform workers in Johannesburg and a reflexive thematic analysis of their accounts, it has argued that legislative exclusion is not a gap awaiting technical correction but a structuring condition with an identifiable shape: the state maintains a definitional boundary around the employment relationship through four identifiable processes, that boundary sustains a jurisdictional space in which platform and client practices operate unconstrained, and those practices are associated with unbounded working time, an unreviewable withdrawal of work, the privatization of social protection, and cumulative exclusion from the institutions of financial and social citizenship. The account holds for both segments of the sample in this general form, but its content differs between them: location-based participants encountered exclusion as an unaccountable platform, and remote online participants as an unenforceable market.

The contribution of the study lies less in the description of these conditions than in the account of how they are produced. Under legislative exclusion, risk is transferred from platforms and clients to workers and their households, algorithmic and contractual control is encountered as authority that cannot be reviewed, and disadvantage is compounded as the documentary routes through which insecure work might otherwise be stabilized are closed off. In a labor market where gig work is a primary source of income and alternatives are scarce, that configuration operates with unusual force.

Three implications follow. For regulation, reform proposals, whether a presumption of employment, an intermediate protected status, enforceable duties of algorithmic transparency, or more consistent application of the presumptions already contained in section 200A of the LRA and section 83A of the BCEA, should be assessed against their capacity to interrupt this configuration rather than against their capacity to address particular symptoms, and against which segment they are capable of reaching. Instruments directed at platforms acting as de facto employers will reach e-hailing and delivery; freelancers and content creators require measures that do not depend on identifying a local employer, such as payment-protection duties on marketplaces and social security detached from employment status. For platforms, transparent governance of suspensions and deactivations, including appeal mechanisms with human review, written reasons for adverse decisions, and tiered warnings, would reduce arbitrariness even in advance of legislative change, and marketplaces mediating online work would achieve a comparable effect through enforceable escrow, published ranking criteria and a route of appeal against demonetization. For workers, the digital networks through which participants already share information about unsafe areas, platform changes and income volatility offer a basis for more structured collective voice, particularly where aligned with established labor movements. The basis for organization differs by segment, however: location-based workers share a city, a workplace of sorts and a common counterparty, whereas online workers share an occupation but neither a location nor an employer, so their collective forms are likely to resemble occupational associations more closely than bargaining units. In neither case can such strategies substitute for statutory protection.

Future research should pursue systematic cross-national comparison between jurisdictions with and without a statutory presumption of employment, which would allow the significance of legislative design to be considered separately from that of labor market structure and examine the account proposed here more directly. Comparison within a single country between the location-based and remote online segments would be at least as valuable, since the two are subject to the same national labor law and differ chiefly in the reach of that law over them, which makes them an unusually well-matched pair for examining what legislative recognition can and cannot do. Longitudinal work would allow examination of whether the conditions described change following legislative recognition, or whether recognition is absorbed as the transport regulations were, as a further set of compliance costs borne by a worker who remains, in practice, an operator. What participants described was not a wish to see platform work abolished, but a claim to protection, to reasons for the decisions that shape their livelihoods, and to a forum in which those decisions can be contested.

## Data Availability

The raw data supporting the conclusions of this article will be made available by the authors, without undue reservation.
